# Can Pin-on-Disk Testing Be Used to Assess the Wear Performance of Retrieved UHMWPE Components for Total Joint Arthroplasty?

**DOI:** 10.1155/2014/581812

**Published:** 2014-09-11

**Authors:** Steven M. Kurtz, Daniel W. MacDonald, Sevi Kocagöz, Mariya Tohfafarosh, Doruk Baykal

**Affiliations:** ^1^Implant Research Center, School of Biomedical Engineering, Science, and Health Systems, Drexel University, 3401 Market Street, Suite 345, Philadelphia, PA 19104, USA; ^2^Exponent, 3440 Market Street, Suite 600, Philadelphia, PA 19104, USA

## Abstract

The objective of this study was to assess the suitability of using multidirectional pin-on-disk (POD) testing to characterize wear behavior of retrieved ultrahigh molecular weight polyethylene (UHMWPE). The POD wear behavior of 25 UHMWPE components, retrieved after 10 years* in vivo*, was compared with 25 that were shelf aged for 10–15 years in their original packaging. Components were gamma sterilized (25–40 kGy) in an air or reduced oxygen (inert) package. 9 mm diameter pins were fabricated from each component and evaluated against CoCr disks using a super-CTPOD with 100 stations under physiologically relevant, multidirectional loading conditions. Bovine serum (20 g/L protein concentration) was used as lubricant. Volumetric wear rates were found to vary based on the aging environment, as well as sterilization environment. Volumetric wear rates were the lowest for the pins in the gamma inert, shelf aged cohort. These results support the utility of using modern, multidirectional POD testing with a physiologic lubricant as a novel method for evaluating wear properties of retrieved UHMWPE components. The data also supported the hypothesis that wear rates of gamma-inert liners were lower than gamma-air liners for both retrieved and shelf aging conditions. However, this difference was not statistically significant for the retrieved condition.

## 1. Introduction

Total joint replacements, which represent the current standard of care for end-stage, degenerative hip and knee disease, typically incorporate a metal or ceramic component articulating against a counterface of ultra-high molecular weight polyethylene (UHMWPE) [1]. The tribological behavior of UHMWPE biomaterials is known to be influenced by their extent of crosslinking, which improves wear resistance [2–5], or by oxidation, which decreases it [6–9]. There is great interest in evaluating changes to the wear behavior of UHMWPE formulations after implantation in the body [8, 10–16].

Pin-on-disk (POD) testers are currently employed as a screening tool for orthopaedic biomaterials, because they can economically identify promising bearing couples before expensive joint simulations are warranted [17]. Although earlier POD testers were not capable of reproducing clinically relevant wear mechanisms, multidirectional POD testers are able to rank the tribological properties of different formulations of clinically relevant ultra-high molecular weight polyethylene (UHMWPE)* in vitro* [17]. Through efficient design, POD testers with up to 100 individual specimen stations are now available [18], which can evaluate a potentially statistically relevant sample size for biotribological simulations.

POD simulation has been successful in evaluating the as-manufactured and* in vitro* aged UHMWPE [17]. However, little is known about the response of* in vivo* aged UHMWPE in POD testers. Oxidation of gamma sterilized UHMWPE occurs after exposure of oxygen in air as well as after implantation in the body [8, 10–16], either of which would theoretically increase wear rate of the material. However, clinical studies of human patients demonstrate the opposite trend; namely, the wear rate of UHMWPE components tends to* decrease* with implantation time [19]. Thus, the extent to which* in vivo* conditions will affect the tribological properties of UHMWPE remains poorly understood. The objective of this study was to assess the suitability of using POD testing to characterize the biotribological behavior of retrieved UHMWPE components for total joint replacement. We also compared the oxidative effects of irradiation in air and in inert gas on the wear resistance of UHMWPE and evaluated the magnitude of these effects on shelf-aged and retrieved implants. We hypothesized that irradiation in inert gas would result in lower wear rates compared to irradiation in air. Our second hypothesis was that retrieved implants would be more wear resistant than shelf-aged implants.

## 2. Materials and Methods

**Figure 1:**
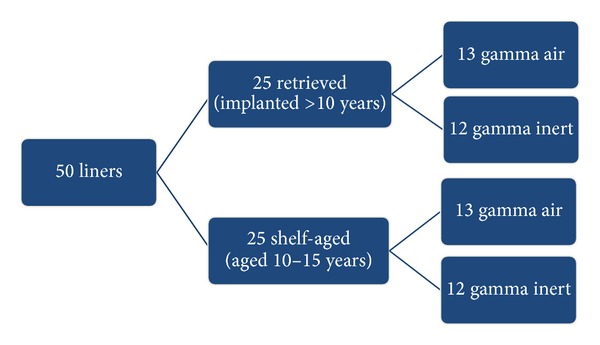
Schematic of the four cohorts for the retrieved and shelf-aged UHMWPE components.

Retrieved (*n* = 25) hip liners that were consolidated from GUR 1050 resin and gamma irradiated with a dose of 25–40 kGy were identified from our center's institutional collection of over 2,000 retrieved devices based on their sterilization method (air versus inert) and implantation time (>10 years). Shelf-aged implants (*n* = 25), which were aged 10–15 years, served as never implanted controls. In each group, 13 implants were irradiated in air and 12 implants were irradiated in inert gas (Figure 1). We selected these four cohorts of UHMWPE materials to analyze because of their extensive track record in the literature [8, 20].

Two cylindrical specimens were produced from each liner using a 9 mm diameter core punch. Of the two cores obtained from the retrieved liners, one core was obtained from the superior half and the second core was obtained from the inferior half (*n* = 2 pins per retrieved liner, 50 pins total). The cores were machined on a lathe to ensure flat surfaces. Thus, the measurements pertain to the wear resistance of material near the surface. For the shelf-aged liners, 1 mm of the surface of one core was removed using a lathe to expose the highly oxidized region whereas the second core was lathed similar to the retrieved cores (*n* = 2 pins per shelf-aged liner, 50 pins total). Specimens were soaked in distilled water for five days before testing.

**Figure 2:**
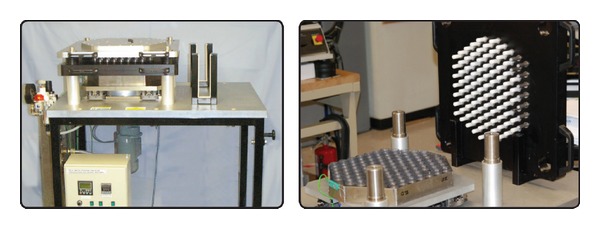
Photographs of the 100-station pin-on-disk tester (load soak station not shown).

Multidirectional POD wear testing in a physiologically relevant lubricant was conducted on a Super-CTPOD with 100 stations (Phoenix Tribology, England) (Figure 2) [18]. The counterface disks were wrought CoCr alloy with an average roughness of 1 ± 0 nm as measured by white light interferometry using a NewView 5000 Model 5032 equipped with advanced texture analysis software, MetroPro 7.7.0 (Zygo, Middlefield, CT). The lubricant used was alpha calf serum with a protein concentration of 20 g/L (Wear Testing Fluid, HyClone, UT). Each pin had its own chamber filled with approximately 15 mL lubricant, which was maintained at 37 ± 1°C. An elliptical wear pattern (major axis 10 mm and minor axis 5 mm) was employed to produce multidirectional wear. The average sliding speed was 24 mm/s. Static loading was applied to generate 2.0 MPa of nominal contact stress.

The testing was carried out to 2.5 million cycles at 1.0 Hz. Gravimetric measurements were carried out at 0, 0.25, 0.50, 1.00, 1.50, 2.00, and 2.50 million cycles using a calibrated digital scale (accuracy = 0.01 mg). At each testing interval, articulating surfaces of pins were photodocumented. The lubricant was replaced after each interval analysis. Five gamma air and five gamma inert pins were employed as load soak control to compensate for the absorption of fluid by pins. To convert mass loss to volumetric loss, 0.931 g/cm^3^ was used as bulk density of GUR 1050 UHMWPE [1]. Wear rates were calculated using a linear regression of volumetric losses.

The distribution of wear rates was tested for normality using the Shapiro-Wilk Test and found to be not normal. Thus, nonparametric statistical testing was used throughout this study. Differences between groups were evaluated with Kruskal-Wallis one-way analysis of variance followed by post hoc Dunn test. Statistical significance was assumed for *P* < 0.05.

## 3. Results

**Figure 3:**
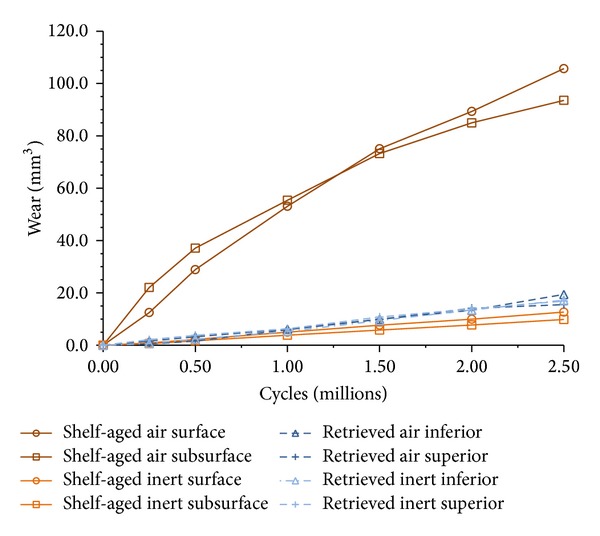
Average volumetric loss versus number of cycles is shown.

**Figure 4:**
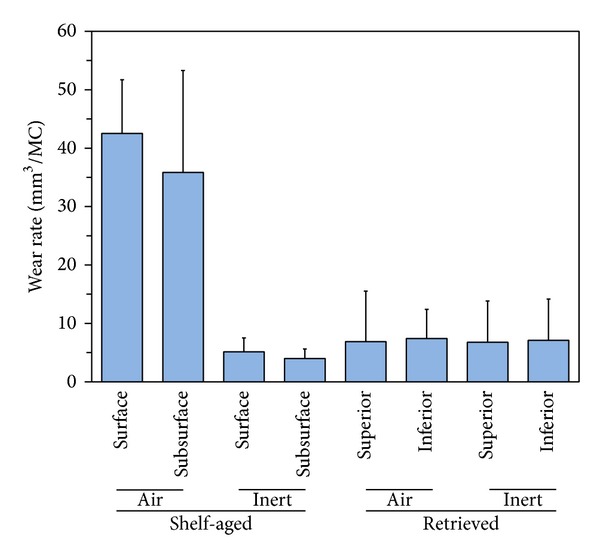
Wear rate of pins grouped by sterilization environment, aging environment, surface/subsurface measurements, and superior/inferior measurements.

**Figure 5:**
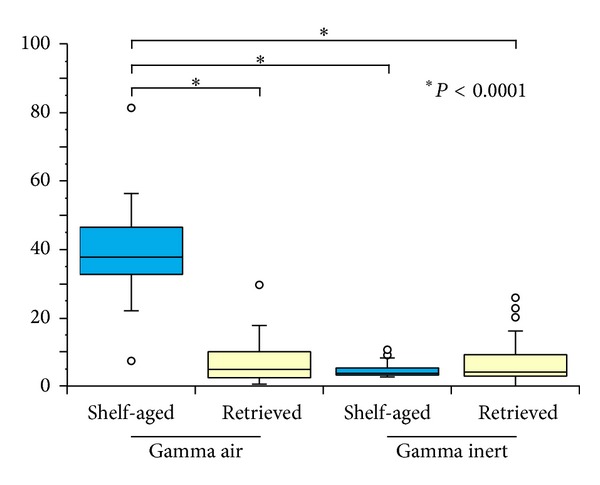
Box plots illustrating the differences in wear rates among the four cohorts. The shelf-aged gamma air pins exhibited the highest wear rates.

Total volumetric loss and volumetric wear rates for pins grouped by sterilization environment, aging environment, surface/subsurface measurements, and superior/inferior measurements are shown in Figures 3 and 4. Volumetric wear rates were found to vary based on the aging environment (i.e., shelf-aged or retrieved,* in vivo*; *P* < 0.0001), as well as sterilization environment (i.e., gamma air or gamma inert; *P* < 0.0001). However, we were unable to detect statistically significant differences in wear rates between surface/subsurface measurements or superior/inferior measurements when controlling for sterilization environment and aging conditions (*P* > 0.05). Therefore, pins were then pooled together based only on aging environment and sterilization environment, and volumetric wear rates were recalculated (Figure 5).

Volumetric wear rates (WR) were highest for the pins in the shelf-aged gamma air cohort (WR = 39.4 ± 13.7 mm^3^/MC; Figure 5; *P* < 0.0001). The retrieved gamma air pins had the next highest wear rates (WR = 7.2 ± 7.0 mm^3^/MC). Although the gamma inert retrievals had lower wear rates (WR = 7.0 ± 6.9 mm^3^/MC), this difference between gamma inert shelf-aged pins and gamma inert retrievals was not statistically significant (*P* = 1.0). The cohort with the least amount of wear was the gamma inert shelf-aged pins (WR = 4.6 ± 2.1 mm^3^/MC).

**Figure 6:**
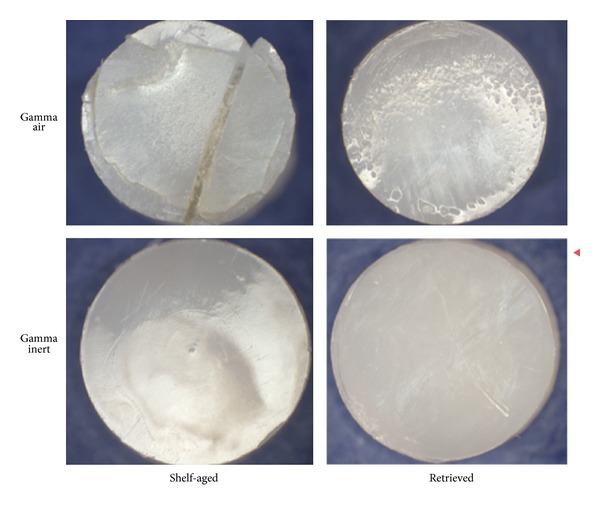
Example pins from the four study cohorts after 0.5 million cycles.

**Figure 7:**
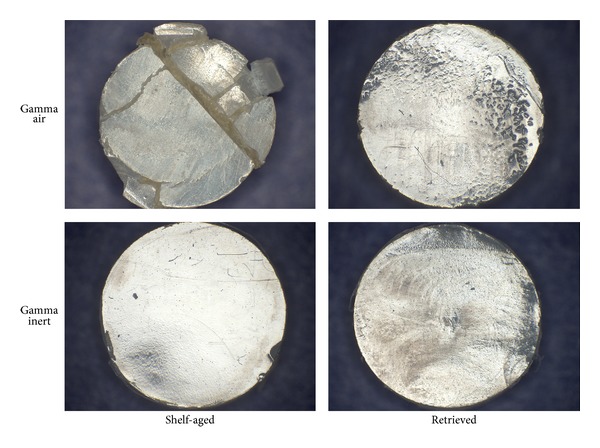
Example pins from the four study cohorts after 2.5 million cycles. Note the extensive cracking in the gamma air, shelf-aged specimen.

The pins of all specimens showed removal of machining marks as well as burnishing of the articulating surface after 0.5 million cycles of testing (Figure 6). The shelf-aged gamma air pins (both surface and subsurface specimens) showed evidence of both surface and subsurface cracking at the end of the wear test after 2.5 million cycles (Figure 7).

## 4. Discussion

The results of this study support the utility of using modern, multidirectional pin-on-disk testing with a physiologic lubricant as a novel method for evaluating the wear properties of retrieved UHMWPE components for total joint replacement, which were exposed to* in vivo* conditions. This study also provides independent verification of the reproducibility of the 100-station POD tester, which has been previously studied by its developer using conventional, never implanted UHMWPE materials [18, 21]. The data also supported the hypothesis that wear rates of gamma inert liners were lower than gamma air liners for both retrieved and shelf aging conditions. However, this difference was not statistically significant for retrieved condition.

We would like to highlight several limitations of this study for the reader. First, the UHMWPE components in this study were obtained from hip arthroplasty. Although we have no reason to suspect that the mechanism of shelf aging would differ between hip and knee arthroplasty devices [20], the results of the explants may not be generalizable to knee retrievals, which may be subjected to higher contact stresses than in hip replacements [11, 12, 14]. Because of the greater radius of curvature, the hip components represent a worst-case scenario for verification of the coring and pin-on-disk testing approach. It is straightforward to extend the methods of the present study from hips to knees given the encouraging findings of the current experiment. Second, the shelf-aged components that were used for this study were all stored in expired packaging, which typically prescribes 5 years for the shelf-life of gamma sterilized components [20]. Again, we selected these long-term shelf-aged components to provide a worst-case, highly oxidized set of control materials to verify the suitability of the pin-on-disc technique. Thus, the data for these shelf-aged components should not be misinterpreted to represent the behavior of materials that would have been used in patients.

Relatively few POD studies have been conducted on specimens obtained from aged UHMWPE biomaterials [22–24]. Besong and colleagues wear-tested UHMWPE pins fabricated from shelf-aged components and observed that gamma irradiation in air, followed by long-term aging (up to 10 years), as well as the counterface roughness, had a dramatic effect on the wear rate [22]. Muratoglu and colleagues conducted POD tests on specimens following accelerated aging conditions [23]. We found only one POD study, which has been conducted on explanted UHMWPE components. Kwon-Yong et al. tested 12 retrieved hip liners on a pin-on-disk tester. There were two fundamental differences between their study and the current study: (1) the retrievals in Kwon-Yong et al. study were shelf-aged in air 1.5–8 years after surgery before they were tested; (2) the pin-on-disk tester in that study did not produce cross-shear. Ultimately, tribometers employed in these previous tests were capable of evaluating up to 6 specimens concurrently, in contrast with the 100-station apparatus in the present study.

The results of this study mirror findings on retrieved UHMWPE liners. Previously, Kurtz et al. found that the femoral head offers protection to the superior surface from oxygen-containing bodily fluids, resulting in lower oxidation [8]. Additionally, previous studies have found that gamma inert packaging has lower oxidation than gamma air packaging [11, 25]. However, since the polyethylenes are identical (except with respect to sterilization environment), once the gamma inert components are removed from their packaging, they will oxidize at similar rates. This is especially apparent when comparing the POD wear rates between the shelf-aged gamma air and gamma inert cohorts. The pins that were shelf-aged in air had POD wear rates that were approximately 8 times greater than the pins that were shelf-aged in a vacuum-sealed inert environment. Therefore, shelf-life and environment are important factors in* in vivo* oxidation, as well as POD wear rates.

The relative oxidative stability of gamma inert UHMWPE, which has been documented in literature [11, 20], probably contributed to the tribological properties measured in this study. Our second hypothesis that shelf-aged UHMWPE would wear more than* in vivo* aged UHMWPE was supported only in the case of gamma air sterilized components. However, gamma inert shelf-aged components had the lowest wear rates of all the components we tested. This suggests that the sterilization in an inert environment, coupled with effective barrier packaging, is capable of preserving the tribological properties of UHMWPE even after storage on the shelf for more than 10 years.

Another important finding of this study was that long-term aging in air after sterilization in air appears to degrade the wear properties of UHMWPE more aggressively than* in vivo*. This may be due, in part, to the femoral head protecting the articulating surface from oxidizing species in body fluids [8]. Our findings for retrieved conventional liners in the present study will be a useful baseline comparison for evaluating the wear properties of retrieved highly crosslinked polyethylene liners, which were exposed to* in vivo* conditions, in future research.
